# From a Shared Stress to Cell‐Type‐Specific Responses: The Heterogeneous Mechanisms of High Glucose‐Induced Cellular Senescence in Diabetic Kidney Disease

**DOI:** 10.1155/jdr/9157351

**Published:** 2026-04-30

**Authors:** Jing Wang, Tian Liu, Kai Zhu, Ni Lin, Qi Wang, Beijia Xu, Xiaomin Wang

**Affiliations:** ^1^ Department of Endocrinology, Hospital of Chengdu University of Traditional Chinese Medicine, Chengdu, Sichuan, China, cdutcm.edu.cn; ^2^ Department of Oncology, Hospital of Chengdu University of Traditional Chinese Medicine, Chengdu, Sichuan, China, cdutcm.edu.cn; ^3^ Chengdu University of Traditional Chinese Medicine, Clinical Medical College, Chengdu, Sichuan, China, cdutcm.edu.cn; ^4^ Chengdu Sport University, School of Sports Medicine and Health, Chengdu, Sichuan, China, cdsu.edu.cn; ^5^ Heilongjiang Academy of Chinese Medicine Sciences, Graduate School, Harbin, Heilongjiang, China, hljtcm.com.cn

**Keywords:** cellular senescence, diabetic kidney disease, glomerular endothelial cell, heterogeneity, mesangial cell, podocyte, renal tubular epithelial cell, senescence-associated secretory phenotype

## Abstract

Diabetic kidney disease (DKD) is a severe microvascular complication of diabetes characterized by complex pathogenesis in which renal cellular senescence is a critical pathological element. Traditionally, hyperglycemia has been regarded as a uniform stressor inducing cellular senescence; however, significant heterogeneity exists in different renal cell types’ responses to high glucose (HG) stimulation. This review systematically elucidates mechanisms underlying senescence of major renal cell types under hyperglycemic conditions. Hyperglycemia acts as a common initiator triggering senescence via shared pathways, including oxidative stress and metabolic dysregulation. However, owing to distinct structural, functional, and molecular profiles across cell types, divergent senescence programs are activated. Specifically, podocyte senescence centers on GSK3*β*‐mediated collapse of metabolic homeostasis and GPR124 axis‐related mechanosensing dysfunction; mesangial cell (MC) senescence manifests as STAT5‐ and Caveolin‐1 signaling‐mediated “senescence–fibrosis” vicious cycles; glomerular endothelial cell (GEC) senescence is characterized by dysregulation of the NOS/NO signaling axis and glycocalyx damage; and renal tubular epithelial cell (TEC) senescence is initiated by mitochondrial damage under metabolic overload, promoting interstitial fibrosis through the senescence‐associated secretory phenotype (SASP). By revealing this heterogeneous mechanism shifting from “common stress” to “specific responses,” this review offers a novel perspective on DKD pathogenesis and establishes a theoretical foundation for developing targeted anti‐senescence therapies. It further discusses implications for the clinical translation of renal protective agents.

**Highlights**

Describes a “shared‐stress‐to‐specific‐response” model of heterogeneous renal senescence in DKD.

Deciphers heterogeneous senescence‐driving networks across distinct renal cell types.

Lays the groundwork for cell‐type‐specific therapies targeting senescent cells.

## 1. Introduction

Diabetic kidney disease (DKD), a major microvascular complication of both Type 1 and Type 2 diabetes, is defined by characteristic pathological structural alterations and progressive renal functional decline under chronic metabolic disturbances such as hyperglycemia, clinically manifesting as persistent proteinuria, hypertension, and progressive glomerular filtration rate (GFR) reduction [1]. DKD is the leading cause of end‐stage renal disease (ESRD) globally [2], with incidence, mortality, and socioeconomic burden steadily increasing over the past two decades, representing a critical public health challenge [3–5]. Current therapies focus primarily on metabolic and hemodynamic control, including rigorous glycemic and blood pressure management, renin–angiotensin system blockade, and nonsteroidal mineralocorticoid receptor (MR) antagonists to delay progression. Novel agents such as sodium‐glucose cotransporter 2 (SGLT2) inhibitors, glucagon‐like peptide‐1 (GLP‐1) receptor agonists, and finerenone have shown efficacy in slowing DKD progression and reducing cardiorenal risk; however, limitations and adverse effects persist [6, 7]. Importantly, these treatments do not fully arrest renal disease progression or significantly reduce complication and mortality risks, resulting in many patients inevitably progressing to ESRD [8, 9]. These limitations highlight fundamental gaps in current treatments targeting classical metabolic and hemodynamic pathways and underscore the urgent need for novel pathophysiological strategies.

Within this context, cellular senescence has attracted growing attention as a key pathophysiological mechanism central to DKD initiation and progression [10]. Notably, cellular senescence is not unique to DKD but represents a nonspecific mechanism shared among chronic kidney diseases, including nondiabetic nephropathies [11, 12]. Substantial evidence demonstrates that the diabetic microenvironment, especially chronic hyperglycemia, induces irreversible senescence cascades in multiple renal cell types, including podocytes, mesangial cells (MCs), glomerular endothelial cells (GECs), and tubular epithelial cells (TECs) [13–15].

The hallmark of cellular senescence is irreversible cell cycle arrest, accompanied by aberrant secretion of diverse pro‐inflammatory cytokines, chemokines, and matrix metalloproteinases, collectively referred to as the senescence‐associated secretory phenotype (SASP) [16]. In human biopsy specimens, senescence is identified through characteristic molecular markers primarily including elevated cyclin‐dependent kinase inhibitors (p16^INK4a^ and p21^CIP1^), enhanced senescence‐associated *β*‐galactosidase (SA‐*β*‐gal) activity, and increased SASP factor expression [17, 18]. These markers distinguish senescent cells from alternative fates such as apoptosis. SASP exacerbates local chronic inflammation and fibrosis via autocrine signaling and induces senescence in neighboring cells through paracrine “bystander effects,” establishing a self‐amplifying pathological vicious cycle [19]. This cascade ultimately disrupts renal structural integrity and leads to progressive functional loss [10]. Consequently, targeting senescent cell clearance (senolytics) or modulating their harmful secretions (senomorphics) has emerged as a promising novel approach for DKD treatment [10, 20].

Importantly, cellular senescence is highly heterogeneous and dynamic [21]. Different cell types exhibit distinct senescence phenotypes due to unique physiological functions, microenvironmental contexts, and varied stress responses. This heterogeneity spans driving mechanisms, molecular expression dynamics, cell‐specific SASP profiles, and differential therapeutic sensitivities [22–24]. Understanding this cell‐type‐specific senescence heterogeneity is vital for elucidating DKD pathogenesis, identifying precise intervention targets, and designing individualized treatments.

Despite considerable research and recent systematic reviews on DKD senescence mechanisms [25], many studies remain limited by viewing senescence as a homogeneous process or focusing on single cell types, lacking systematic comparative analyses of heterogeneous senescence across renal cell types. This gap impairs comprehensive understanding of DKD’s complexity and limits translation of basic research into precise clinical therapies.

While this review emphasizes mechanisms directly induced by hyperglycemia, it is essential to acknowledge that DKD progression is often accelerated by systemic factors, notably hypertension. Hypertension elevates glomerular capillary pressure and mechanical stress, synergistically exacerbating renal cell injury, senescence, and fibrosis, thereby compounding damage initiated by metabolic derangements [26, 27].

Recognizing the synergistic roles of hemodynamic factors like hypertension, this review focuses specifically on hyperglycemia as the primary metabolic driver. It systematically elucidates how hyperglycemia induces heterogeneous senescence responses across podocytes, TECs, MCs, and GECs in DKD. A thorough comparison of dominant driving mechanisms, molecular signatures, and SASP profiles among these cell types aims to isolate confounding influences and clarify direct pathological pathways. This approach addresses existing knowledge gaps and establishes a theoretical basis for understanding DKD pathogenesis and developing precise anti‐senescence therapies tailored to cell types.

## 2. The Hyperglycemic Microenvironment: A Common Origin and Differential Driver of Renal Cellular Senescence Heterogeneity

Chronic hyperglycemia in diabetes not only constitutes a primary driver of renal injury but also extends its pathological impact beyond classical glucotoxicity, acting as a key trigger of accelerated senescence across multiple renal parenchymal cell types [28]. Persistent hyperglycemic conditions profoundly disrupt renal cellular homeostasis through a complex network of direct and indirect molecular events, ultimately leading cells into irreversible senescence [29]. Understanding this critical microenvironmental driver is fundamental for unraveling the heterogeneity of renal cellular senescence in DKD.

**Figure 1:**
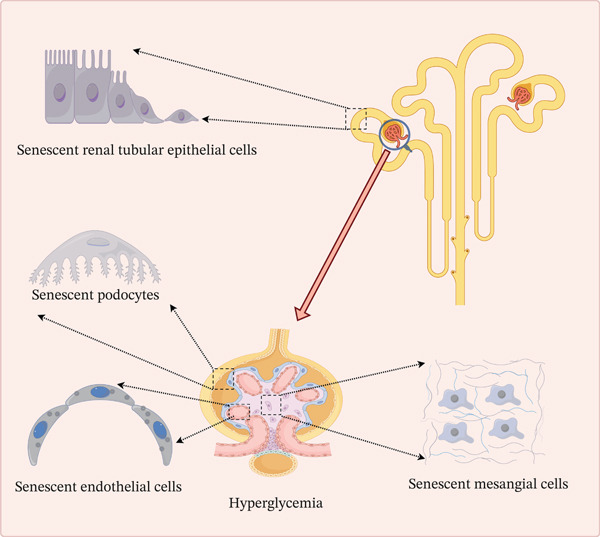
Hyperglycemic microenvironment induces senescence in major renal cell types (the figure was generated with the assistance of Figdraw (http://www.figdraw.com/).

Throughout DKD progression, key renal cell populations responsible for maintaining structural and functional kidney integrity are particularly vulnerable to hyperglycemia‐induced senescence [29]. Podocytes, as essential terminally differentiated components of the glomerular filtration barrier, underpin proteinuria development when their numbers decrease or structures are compromised [30]. GECs form the filtration barrier and regulate vascular tone and inflammatory responses [31]. MCs, constituting the glomerular matrix scaffold, drive glomerulosclerosis through aberrant activation, proliferation, and extracellular matrix (ECM) deposition [32]. TECs, executing reabsorptive and secretory functions, are pivotal in tubulointerstitial fibrosis and progressive renal dysfunction [33]. Notably, tubulointerstitial injury arises both from direct hyperglycemia‐induced metabolic dysregulation and secondary vascular and glomerular injury. Glomerular capillary damage and endothelial dysfunction induce tubular ischemia, hypoxia, and inflammatory microenvironments that accelerate TEC senescence and fibrosis [34]. Collectively, these four cell types represent central players and primary targets for senescence within the hyperglycemic environment of DKD (Figure 1).

Hyperglycemia‐induced cellular senescence involves multiple interconnected molecular mechanisms that synergistically comprise a complex pathogenic network. High glucose (HG) induces mitochondrial dysfunction and activates NADPH oxidases, resulting in marked reactive oxygen species (ROS) overproduction. Elevated ROS surpass endogenous antioxidant defenses, causing oxidative damage to biomolecules such as DNA [35]. Simultaneously, hyperglycemia activates inflammatory pathways including NF‐*κ*B and JAK/STAT, promoting abundant pro‐inflammatory factor production [36], with chronic inflammation serving as a potent senescence inducer [37]. HG also directly impairs mitochondrial structure/function, reducing ATP synthesis, increasing ROS leakage, disrupting calcium homeostasis, and diminishing mitophagy [38]. Accumulated ROS cause extensive DNA damage [39], while HG impairs DNA repair capacity [40], leading to persistent DNA damage response (DDR) activation and subsequent senescence induction via core effectors like p53 [41]. Mitochondrial dysfunction and endoplasmic reticulum stress (ERS) synergistically activate the unfolded protein response (UPR); sustained ERS also promotes senescence [42, 43]. Moreover, hyperglycemia accelerates generation of advanced glycation end products (AGEs), which, through receptor for AGEs (RAGE) engagement, further stimulate oxidative stress and inflammation [44], directly inducing renal senescence and amplifying the cascade, including accelerated telomere shortening [29, 45].

These mechanisms operate synergistically and reinforce each other rather than acting independently. Variations in tissue architecture, functional properties, stress defense abilities, and signaling networks cause pronounced heterogeneity in renal cell types’ sensitivity and reliance on these senescence drivers. For example, podocytes may be particularly sensitive to oxidative stress, whereas MCs primarily depend on TGF‐*β* signaling. This cell type‐specific mechanistic disparity forms the pathological complexity core in DKD and guides future targeted anti‐senescence therapies’ design and implementation.

## 3. Heterogeneous Mechanisms of Renal Cellular Senescence: From Shared Stresses to Specific Responses

As outlined, hyperglycemia drives renal cellular senescence through an intertwined network of mechanisms. However, differences in tissue architecture, functional properties, stress defense, and signaling capacities result in pronounced heterogeneity in renal cell types’ sensitivity and reliance on these pro‐senescence pathways. This section systematically details hyperglycemia‐induced senescence mechanisms in podocytes, MCs, GECs, and TECs, comparing their distinct driving mechanisms, key signaling nodes, and SASP characteristics to establish a molecular basis for precise therapeutic strategies.

### 3.1. Podocyte Senescence: Collapse of Metabolic Homeostasis and Dysregulation of Mechanosensing

**Figure 2:**
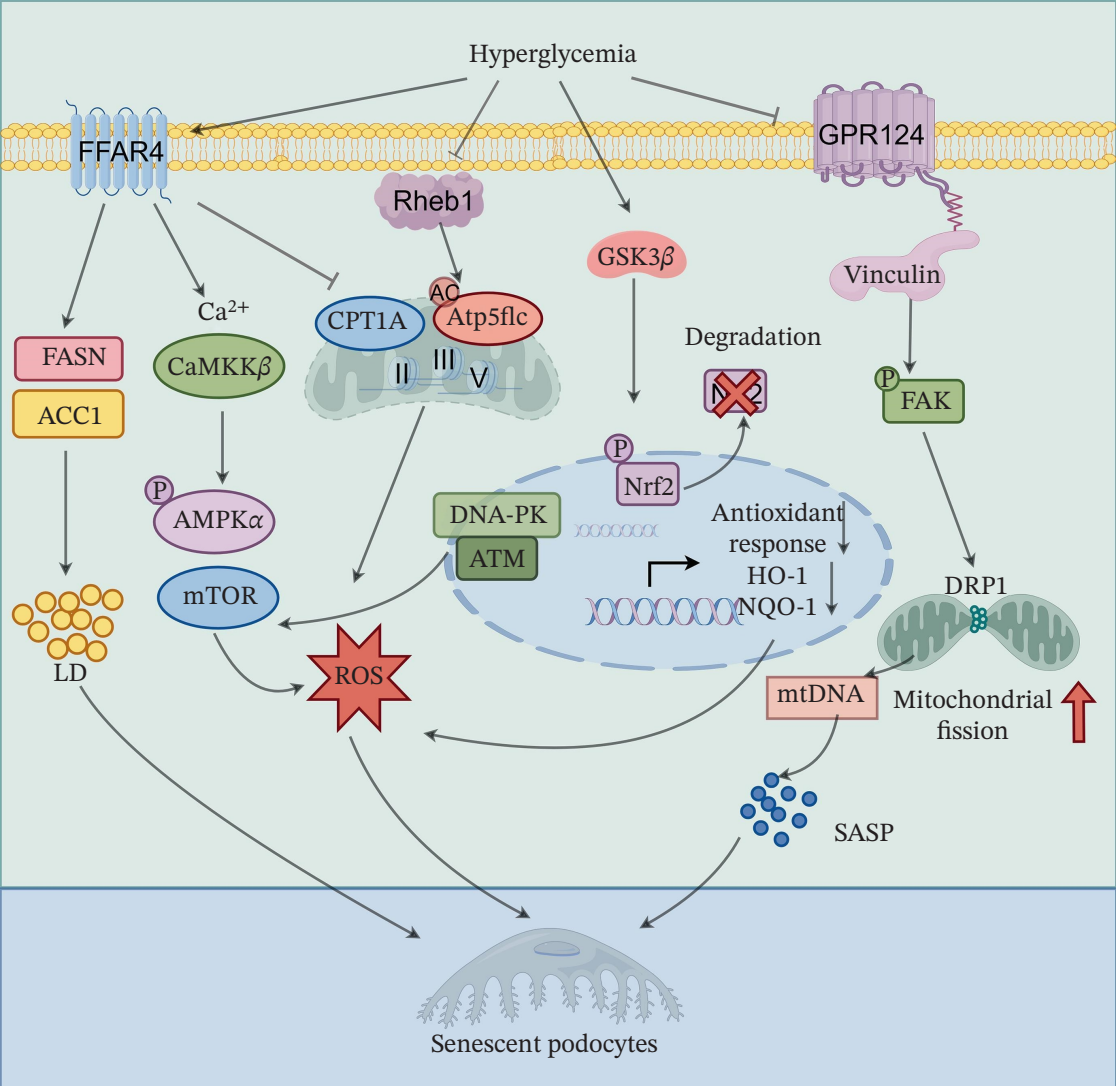
Core mechanisms of high glucose‐induced podocyte senescence.

As central components of the glomerular filtration barrier [46], podocytes possess unique biological traits conferring inherent susceptibility to hyperglycemia‐induced senescence, exhibiting distinct underlying mechanisms (Figure 2).

The HG microenvironment drives podocyte senescence through three interacting pathways. **Metabolic crisis:** HG inhibits FFAR4/Rheb1, inducing lipotoxicity and energy exhaustion, which consequently triggers a burst of ROS. **Oxidative stress integration:** GSK3*β* activation inhibits the Nrf2 defense system, amplifying oxidative stress signals and directly activating the p53/p16‐Rb senescence axis. **Mechanosensing dysregulation:** GPR124 deficiency leads to aberrant FAK activation, resulting in DRP1 phosphorylation‐mediated mitochondrial fragmentation and mtDNA leakage, which in turn activates the inflammatory SASP. These pathways synergistically contribute to the irreversible arrest of the cell cycle and loss of function. The figure was generated with the assistance of Figdraw (http://www.figdraw.com/).

#### 3.1.1. Intrinsic Vulnerability: Structural and Functional Properties Underlying Susceptibility to Senescence

The highly specialized architecture of podocytes underpins both their function and fragility. Interdigitated foot processes (FPs) cover the glomerular basement membrane (GBM), establishing the essential slit diaphragm (SD) filtration barrier [47, 48]. Our prior description of the podocyte structure emphasized its significance; nonetheless, to enhance clarity and avoid redundancy, we briefly summarize key features here. Maintaining this complex architecture and barrier functionality depends on a sophisticated protein network—such as integrin *α*3*β*1 anchoring the cytoskeleton to the GBM and junctional proteins stabilizing the SD complex—distributed across distinct domains including the apical membrane, cytoskeleton, SD, and basal adhesion zones, along with continuous high‐energy supply [49, 50].

As terminally differentiated cells, podocytes exhibit minimal regenerative capacity, residing in G0‐phase cell cycle arrest [51]. This, coupled with limited repair ability, makes them prone to senescence and a primary target of apoptosis‐induced irreversible loss in early DKD [52]. It is critical to distinguish senescence versus apoptosis as distinct terminal fates: senescence is characterized by upregulation of cyclin‐dependent kinase inhibitors (p16, p21), increased SA‐*β*‐gal activity, and sustained SASP secretion; apoptosis involves caspase‐3 activation and DNA fragmentation [53, 54].

Further, podocytes’ high energy demand renders them highly sensitive to metabolic disturbances, while their DNA repair capacity is limited, exemplified by downregulated key repair genes like ERCC1 [55]. Collectively, terminal differentiation, complex architecture, elevated metabolic requirements, and genomic maintenance challenges define podocyte vulnerability in hyperglycemia [49, 56]. When HG‐induced damage surpasses reparative thresholds, podocytes readily advance to senescence and dysfunction. In DKD, podocyte senescence associates with foot process effacement, contributing to proteinuria development [57].

#### 3.1.2. Core Metabolic Drivers: Lipotoxic Energy Crisis, Dysregulated Metabolic Sensing, and Oxidative Stress Burst

Within the DKD hyperglycemic milieu, podocyte metabolic homeostasis is severely disrupted, triggering an intertwined lipotoxic energy crisis and dysregulated metabolic signaling that drive senescence. HG suppresses free fatty acid receptor 4 (FFAR4), impairing protective omega‐3 fatty acid signaling. FFAR4 deficiency upregulates lipogenic enzymes (FASN, ACC1) and downregulates fatty acid oxidation (FAO) factor CPT1*α*, causing lipid droplet accumulation and lipotoxicity [58]. Concurrently, HG downregulates Rheb1, a vital podocyte GTPase; Rheb1 loss induces aberrant acetylation of mitochondrial ATP synthase subunit Atp5f1c and impairs respiratory chain complexes II, III, and V, leading to decreased mitochondrial membrane potential (*ΔΨ*m), severe energy depletion, and ROS overproduction [13].

Lipotoxicity and energy crisis synergize into a vicious cycle: lipotoxicity damages mitochondria, exacerbating energy deficit and ROS generation; energy crisis impairs lipid metabolism, worsening lipid accumulation [59, 60]. This cycle is regulated by metabolic hubs AMPK and mTOR pathways. FFAR4 deficiency inhibits the Ca2+/CaMKK*β*‐AMPK axis, reducing autophagy and heightening lipid dysregulation [58]. DNA damage kinases (DNA‐PK, ATM) activate mTORC1, suppressing autophagy, impairing DNA repair, and promoting aberrant growth signaling [55]. AMPK suppression and mTORC1 activation collectively undermine podocyte maintenance, amplifying metabolic stress and ROS.

In summary, metabolic disruptions and their dysregulated regulatory crosstalk in podocytes under DKD hyperglycemia constitute central drivers activating downstream senescence pathways [55, 58].

#### 3.1.3. The Key Signaling Hub: GSK3*β* Integrates Oxidative Stress to Drive the Core Senescence Pathway

Glycogen synthase kinase 3*β* (GSK3*β*) is markedly enriched and hyperactivated in DKD glomerular podocytes, serving as a central node integrating oxidative stress to drive senescence via dual mechanisms [61, 62].

HG‐enhanced GSK3*β* activity phosphorylates the antioxidant transcription factor Nrf2, limiting its nuclear translocation and decreasing antioxidants such as HO‐1 and NQO1, leading to excessive ROS accumulation. This ROS further activates GSK3*β*, establishing a positive feedback loop. Sustained oxidative bursts induce DNA damage and protein tyrosine nitration, contributing to senescence [63, 64]. Simultaneously, GSK3*β* activates core senescence pathways by phosphorylating and upregulating p16^INK4a^ and p53, which induce retinoblastoma protein (Rb) dephosphorylation. Hypophosphorylated Rb binds and inhibits E2F transcription factors, enforcing irreversible cell cycle arrest characteristic of senescence [63, 65]. Inhibition of GSK3*β* alleviates oxidative stress, restores Nrf2 activity, delays p53/p16 pathway activation and senescence onset, and ameliorates DKD pathology [64].

Therefore, GSK3*β* functions not only as an oxidative stress amplifier but also as a pivotal molecular switch activating downstream senescence (p53/p16‐Rb), linking upstream injury to irreversible podocyte senescence and representing a promising therapeutic target.

#### 3.1.4. Loss of Structural–Mechanical Integrity: GPR124 Axis Disruption Couples Mitochondrial Dysfunction to the Senescence Program

The adhesion G protein‐coupled receptor GPR124 is highly expressed in podocytes, regulating cell polarity and cell‐matrix adhesion [57, 66]—functions critical for maintaining foot process polarity and stable adhesion to the GBM [67]. However, GPR124 expression is markedly downregulated in patients with DKD and experimental models [57].

GPR124 deficiency may drive podocyte senescence via a three‐pronged cascade: loss of interaction with focal adhesion protein Vinculin leads to focal adhesion kinase (FAK) hyperphosphorylation, causing aberrant focal adhesion accumulation and impaired mechanosensing—key steps in foot process effacement and filtration barrier disruption [57]. Aberrant FAK activation phosphorylates mitochondrial fission protein DRP1, inducing excessive mitochondrial fission and fragmentation. Fragmented mitochondria release mitochondrial DNA, activating the cGAS‐STING innate immune pathway and promoting pro‐inflammatory SASP factors such as IL‐6 and TNF‐*α* [51, 57]. Loss of GPR124‐mediated protective signaling removes inhibitory control over core senescence pathways (p53/p21/p16), synergistically accelerating cell cycle arrest and senescence marker accumulation [51, 57].

Thus, the GPR124‐Vinculin‐FAK axis constitutes a critical mechanism linking podocyte structural integrity loss with mitochondrial dysfunction, inflammatory SASP secretion, and activation of core senescence pathways, representing a key driver of podocyte‐specific senescence in response to hyperglycemia and mechanical stress.

### 3.2. MC Senescence: Pathological Metabolic Reprogramming and the “Senescence–Fibrosis” Vicious Cycle

**Figure 3:**
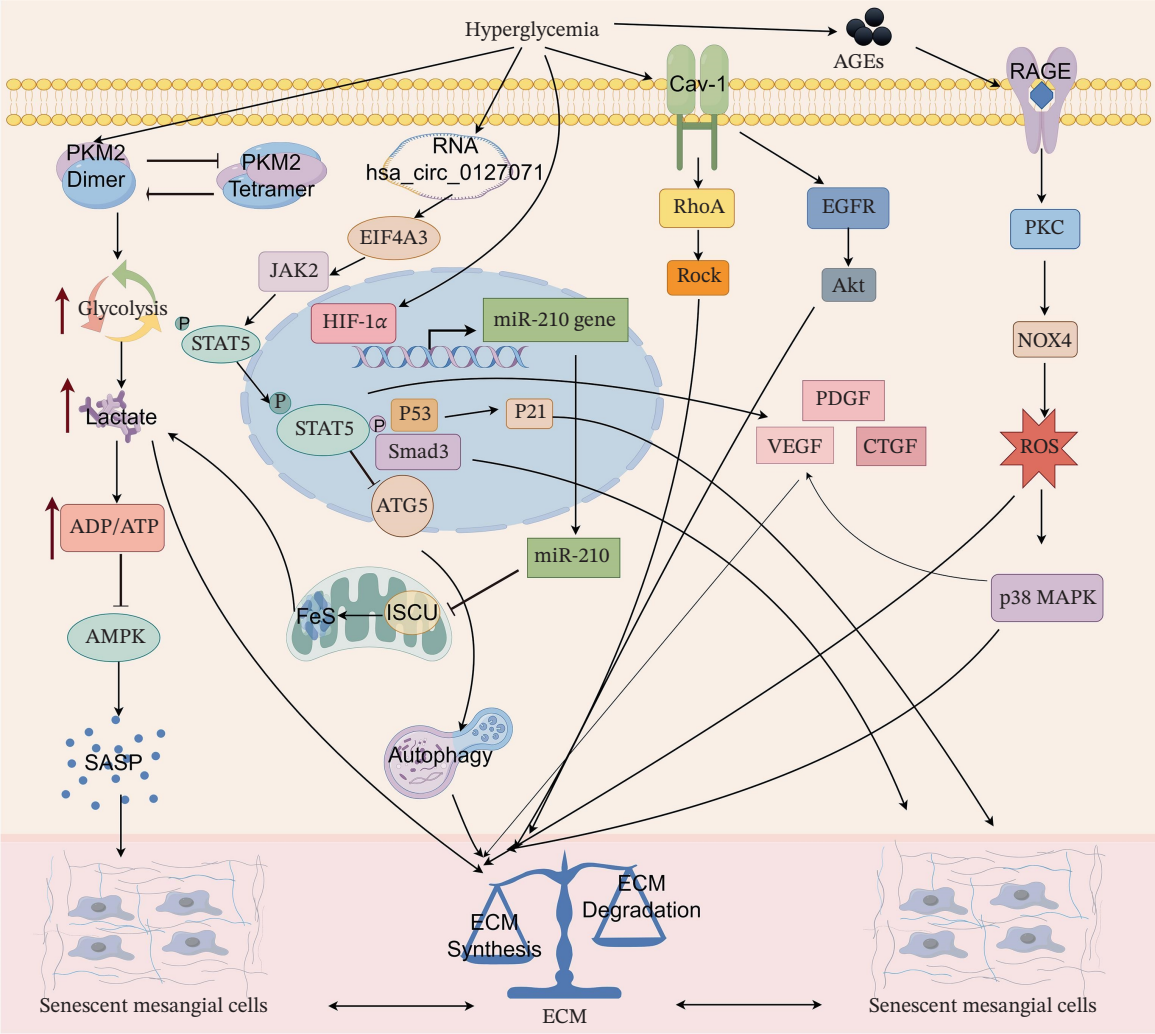
Core mechanisms of high glucose‐induced mesangial cell senescence and fibrosis.

MCs, central to glomerular matrix homeostasis and mechanical stress regulation [68, 69], regulate GFR via contractility, secrete and degrade ECM for structural maintenance, exhibit phagocytic activity to clear trapped macromolecules, and sense hemodynamic signals [70]. These multifunctional roles underpin a susceptibility to hyperglycemia‐induced senescence distinct from other glomerular cells, characterized by pathological matrix feedback loops driven by metabolic disturbance, oxidative stress, and chronic inflammation (Figure 3).

The HG microenvironment drives MC senescence and fibrosis through four interconnected mechanisms. **Pathological metabolic reprogramming:** HG shifts MC metabolism toward glycolysis via the HIF‐1*α*/miR‐210/ISCU/FeS axis, causing lactate accumulation, intracellular acidosis, and AMPK suppression, thereby directly inducing senescence. **NOX4-mediated oxidative stress:** HG upregulates NOX4 via the RAGE/PKC pathway, triggering a burst of ROS that activates p38 MAPK and damages the ECM. **STAT5 signaling hub:** AGEs/RAGE and circular RNA activate the JAK2/STAT5 axis. Nuclear STAT5 cooperates with Smad3/p53 to upregulate p16/p21, inducing cell cycle arrest, suppressing autophagy, and driving pathological ECM transformation. **Caveolin-1-mediated mechano-metabolic integration:** Cav‐1 responds to HG, mechanical stress, and Ang II by activating the RhoA/ROCK and EGFR/Akt pathways, thereby coupling mechanical stimuli to metabolic and oxidative stress responses. These modules form a pathological matrix feedback loop: aberrant ECM amplifies fibrotic signaling, which in turn promotes senescence, creating a self‐perpetuating “senescence–fibrosis” vicious cycle. The figure was generated with the assistance of Figdraw (http://www.figdraw.com/).

#### 3.2.1. Functional and Positional Basis of Vulnerability

MCs reside centrally within glomerular capillary loops, with cytoplasmic processes linked to the capillary basement membrane, forming the glomerulus’s structural scaffold [32]. Their core functions—a contractile response mediated by *α*‐smooth muscle actin (*α*‐SMA) regulating filtration rate, and active ECM synthesis/degradation to maintain basement membrane homeostasis—constitute their intrinsic vulnerability to DKD injury [71, 72].

Although MCs have moderate proliferation and renewal capacity, chronic exposure to HG conditions exhausts this renewal ability [73]. HG disrupts ECM homeostasis by increasing synthesis of collagen IV and fibronectin, leading to mesangial expansion—a key early DKD lesion—creating a fibrotic microenvironment that perpetuates MC and adjacent cell dysfunction and senescence [32]. High expression of TGF‐*β* receptor and integrin *α*v*β*3 sensitizes MCs to pro‐fibrotic and inflammatory stimuli, lacking intrinsic protection and amplifying injury [32, 74]. Furthermore, MCs’ anatomical position exposes them directly to glomerular filtrate components and hemodynamic stress, providing an additional vulnerability source [75].

Consequently, MCs’ core biological functions, high sensitivity to pathological stimuli, and chronic injurious microenvironment collectively promote their accelerated senescence and vulnerability in DKD.

#### 3.2.2. Core Pathological Driving Mechanisms: Metabolic Reprogramming, Oxidative Stress‐Inflammation Cascade, and Pathological Matrix Feedback Loops

HG–induced metabolic reprogramming initiates MC senescence. HG shifts MC metabolism from mitochondrial oxidative phosphorylation to glycolysis via the HIF‐1*α*/miR‐210/ISCU/FeS axis [76]. This aberrant metabolic shift causes lactate accumulation and intracellular acidosis, impairing lysosomal protease activity and ECM degradation [77, 78]. Additionally, dysregulated pyruvate kinase M2 (PKM2) dimer‐tetramer equilibrium exacerbates glycolytic flux imbalance [79], altering ATP/ADP ratios and suppressing AMPK activity, thereby enhancing pro‐inflammatory SASP production and directly driving senescence [80].

Oxidative stress also critically drives MC senescence. Under HG conditions, NOX4, a NADPH oxidase isoform, is specifically overexpressed in MCs, becoming the primary ROS source in DKD. HG elevates NOX4 via the RAGE/PKC signaling pathway, producing a rapid ROS burst [81]. Unlike podocytes, MC NOX4‐derived ROS preferentially target ECM synthesis and degradation, amplifying injury through p38 MAPK pathway activation [82]. This oxidative stress connects metabolic dysfunction with pathological alterations.

Activated MCs secrete pro‐inflammatory cytokines (TNF‐*α*, IL‐6, IL‐8, IL‐1) and pro‐fibrotic factors (TGF‐*β*1, VEGFA, CTGF) [83–85]. These, combined with aberrant ECM produced by MCs, form a pathological matrix feedback loop: autocrine TGF‐*β*1 stimulates MCs further, enhancing NOX4 expression and ECM deposition [85]. Abnormal matrix accumulation activates the FAK‐PI3K signaling axis via integrin *α*v*β*3, potentiating pro‐fibrotic signaling [86].

Therefore, from metabolic reprogramming to NOX4‐mediated oxidative damage, inflammatory factor release, and pathological ECM deposition, a self‐reinforcing cascade forms, driving MC senescence with high‐intensity pathological signaling.

#### 3.2.3. The STAT5 Signaling Axis: A Critical Hub Coupling Senescence With Fibrosis

Within MC senescence’s complex network, the JAK2/STAT5 signaling axis acts as a central coordinating hub with multiple upstream activators. AGEs induce STAT5 phosphorylation via RAGE and enhance JAK2 translation by stabilizing EIF4A3 through circular RNA hsa_circ_0127071, synergistically promoting STAT5 activation [87].

Upon activation and nuclear translocation, STAT5 exerts multiple pro‐senescence effects: (i) forms complexes with Smad3 and p53, upregulating cell cycle inhibitors p16^INK4a^ and p21^CIP1^, enforcing MC cell cycle exit and senescence‐associated arrest [88]; (ii) suppresses ATG5 expression, a key autophagy gene, impairing LC3‐II degradation and autophagosome formation, thereby reducing MCs’ capacity to clear damaged components and accelerating senescence [87].

Critically, STAT5 activation exhibits a positive correlation and functional synergy with TGF‐*β* pathway activity [89]. The chronic HG environment directly activates STAT5, which subsequently upregulates expression of potent pro‐fibrotic growth factors including PDGF, VEGF, and CTGF. This cascade promotes excessive pathological ECM deposition and facilitates myofibroblast transdifferentiation [90]. Importantly, the resulting fibrotic microenvironment becomes a significant driver of cellular senescence, establishing a self‐perpetuating vicious cycle of “senescence–fibrosis” [91].

Thus, aberrant STAT5 activation represents a critical molecular node linking MC senescence, dysfunction, and pathological ECM remodeling in DKD. Targeted STAT5 inhibition shows promise not only for delaying MC senescence but also for effectively blocking pro‐fibrotic cascades, offering a novel strategic approach for interrupting the vicious cycle of glomerulosclerosis in DKD.

#### 3.2.4. Caveolin‐1: The Mechano‐Metabolic Signaling Integrator

Beyond metabolic, inflammatory, and fibrotic signaling pathways, MCs—as central structural support cells within the glomerular capillary tuft—are constantly subjected to mechanical loads including high hydrostatic pressure and shear stress. These mechanical forces represent critical drivers of their senescence process [75]. Within this context, Caveolin‐1 (Cav‐1), a key integral membrane protein and primary structural component of cholesterol‐enriched membrane microdomains (caveolae), serves as a pivotal signaling hub in MCs. Cav‐1 participates not only in cell adhesion, migration, substance transport, and endocytosis but also exhibits significant mechanosensory functions, positioning it as a key molecule linking mechanical stimuli to intracellular signal transduction [92–94].

The sustained HG environment upregulates Cav‐1 protein expression in MCs. Its activation directly induces typical senescent phenotypes—including telomere shortening, ROS burst, and increased SA‐*β*‐gal activity—via the p53/p21 signaling axis [94]. More significantly, functioning as an integrator of mechanical, metabolic, and hormonal signals, Cav‐1 coordinately responds to diverse pathological stimuli. These include mechanical forces such as glomerular hypertension, metabolic/hormonal signals mediated by angiotensin II (Ang II) binding via AT1 receptors, TGF‐*β*1 cytokine signaling, and ROS‐induced oxidative stress. Through activation of the RhoA/ROCK pathway and EGFR/Akt‐mediated aberrant ECM synthesis, Cav‐1 tightly couples mechanical signals with metabolic disturbances, inflammation, and oxidative stress [94, 95]. This integration generates a mechano‐metabolic vicious cycle: mechanical stimuli exacerbate metabolism‐ and oxidative stress‐related damage via Cav‐1, and these damages (e.g., increased ECM deposition, altered vascular tension) subsequently further alter the intraglomerular mechanical environment, establishing a positive feedback loop that collectively drives MC senescence [94].

### 3.3. Senescence of GECs: A Core Driver of Vascular Barrier Disruption and Microcirculatory Dysfunction

**Figure 4:**
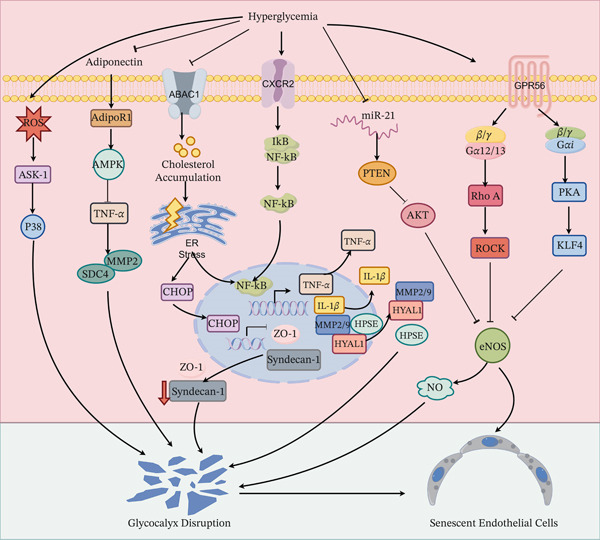
Core mechanisms of high glucose‐induced glomerular endothelial cell senescence.

As the first line of defense of the glomerular filtration barrier and a central regulator of microcirculation, GECs—with their unique fenestrated structure and high metabolic‐hemodynamic sensitivity—become a convergence point for multiple injurious signals under HG conditions [96]. Distinct from podocytes that rely on intricate structures to maintain the filtration barrier, GEC senescence is characterized by disrupted vascular homeostasis: HG‐induced metabolic disturbances, oxidative stress bursts, and aberrant hemodynamic shear stress collaboratively trigger endothelial dysfunction. This ultimately leads to loss of fenestral barrier integrity, capillary hardening (sclerosis), and microcirculatory impairment, establishing GEC senescence as a key driver of proteinuria and glomerulosclerosis progression in DKD [31, 97] (Figure 4).

The hyperglycemic microenvironment synergistically drives GEC senescence through two core pathways. **Glycocalyx Damage and Signal Amplification:** HG induces severe disintegration of the eGlx via multiple mechanisms. Firstly, ROS activates the ASK1‐p38 pathway, directly disrupting eGlx structure. Concurrently, HG levels upregulate the expression of degrading enzymes, such as MMP2/9, HPSE, and HYAL1, through the CXCR2‐NF‐*κ*B axis. Furthermore, the resulting ABCA1 dysfunction inhibits the synthesis of syndecan‐1 and ZO‐1 via the ERS‐CHOP/NF‐*κ*B axis. Additionally, HG impairs the antagonistic effect of adiponectin, mediated by the AdipoR1‐AMPK pathway, against TNF‐*α*‐induced SDC4 and MMP2. The shed eGlx fragments act as DAMPs, inducing cell cycle arrest and propagating senescence signals in a paracrine manner. **eNOS/NO Signaling Axis Dysregulation:** HG suppresses eNOS activity through multiple mechanisms, including the GPR56‐RhoA, GPR56‐PKA‐KLF4, and miR‐21‐PTEN‐AKT pathways. The consequent reduction in NO bioavailability activates the p53/p21 pathway, thereby driving cellular senescence. The figure was generated with the assistance of Figdraw (http://www.figdraw.com/).

#### 3.3.1. Structural Vulnerability and High‐Risk Microenvironment Exposure: The Pathological Basis of Senescence

The fenestrated structure of GECs is covered by a negatively charged endothelial glycocalyx (eGlx) layer, and its diaphragm‐free, hourglass‐like morphology is essential for maintaining the high‐flux filtration characteristic of the glomerulus [98]. However, this structural integrity highly depends on sustained VEGFA signaling and its regulatory proteins [99]. As cells directly lining the glomerular capillary lumen, GECs are continuously exposed to a high‐risk microenvironment—subjected to direct assault from high hemodynamic stress, chronic hyperglycemia, AGEs, and inflammatory factors [100, 101].

This unique anatomical positioning and structural signaling dependency render GECs highly susceptible to injury. HG and its byproducts reduce fenestral density, promote glycocalyx shedding, and induce pathological diaphragm formation. These alterations significantly diminish the effective filtration area and increase vascular resistance, highlighting their inherent structural vulnerability [102]. The combination of structural vulnerability and exposure to a high‐risk microenvironment (characterized by high shear stress, hyperglycemia, and toxins) constitutes the core pathological basis for GEC senescence.

#### 3.3.2. Glycocalyx Injury: Synergistic Driving of Senescence Through Structural Disruption and Signaling Dysregulation

In DKD, eGlx damage represents a pivotal event in endothelial dysfunction, primarily driven by synergistic effects of oxidative stress, inflammatory cascades, lipid metabolism disorders, and hemodynamic alterations. HG‐induced ROS bursts activate the ASK‐1‐p38 pathway, which disrupts eGlx structural integrity via phosphorylation and triggers VE‐cadherin internalization and degradation, leading to physical eGlx shedding [103]. Concurrently, within the inflammatory cascade, CXCR2 activation stimulates NF‐*κ*B signaling, driving TNF‐*α* and IL‐1*β* release and specifically upregulating expression of glycocalyx‐degrading enzymes including MMP2/9, heparanase (HPSE), and hyaluronidase 1 (HYAL1). This establishes a self‐reinforcing cycle of “inflammation → glycocalyx degradation → fragment release → amplified inflammation” [104].

Lipid metabolic dysregulation further exacerbates injury: ABCA1 dysfunction leads to intracellular cholesterol accumulation in GECs, inducing ERS and activating the CHOP/NF‐*κ*B axis. This not only suppresses synthesis of key glycocalyx components such as syndecan‐1 and tight junction protein ZO‐1 but also promotes a pro‐inflammatory microenvironment [105]. Furthermore, reduced adiponectin levels weaken its antagonistic effect—mediated via the AdipoR1‐AMPK pathway—against TNF‐*α*‐induced glycocalyx‐degrading enzymes such as syndecan‐4 (SDC4) and matrix metalloproteinase 2 (MMP2) [106], thereby accelerating early endothelial injury. Additionally, abnormal local shear stress resulting from decreased fenestral density and pathological diaphragmation directly impairs eGlx barrier function and its structural coupling with the endothelial cytoskeleton [107].

The aforementioned multi‐pathway injuries reduce eGlx synthesis and increase shedding, collectively inducing sustained oxidative stress and inflammatory responses that ultimately accelerate the senescent phenotype in GECs [29, 97]. Crucially, shed eGlx fragments function as damage‐associated molecular patterns (DAMPs). By activating pattern recognition receptors, these fragments initiate the p53‐p21 pathway to induce cell cycle arrest and upregulate senescence marker SA‐*β*‐gal activity; simultaneously, they trigger SASP, releasing factors such as IL‐6, IL‐1*β*, and TGF‐*β*. These factors propagate senescence signals paracrinely and amplify the senescent state of the microenvironment [103, 104], forming a vicious cycle progressing from structural damage to functional failure.

#### 3.3.3. A Core Driver of Glomerular Endothelial Senescence: Dysregulation of the NOS/NO Signaling Axis

Nitric oxide (NO), derived from endothelial nitric oxide synthase (eNOS), represents a central molecule maintaining the integrity of the fenestrated structure and hemodynamic barrier function of GECs. It exerts protective effects by regulating vasodilation, anticoagulation, and permeability homeostasis [108–110]. The high dependency on this pathway, combined with GECs’ characteristic as the first line of the filtration barrier—continuously subjected to high perfusion pressure and directly exposed to a high‐risk microenvironment comprising HG, AGEs, and uremic toxins [100, 101, 110]—renders eNOS dysfunction a specific hallmark of GEC senescence.

However, during DKD progression, this pathway encounters multiple synergistic inhibitory mechanisms. The HG environment induces GPR56 expression, which activates the G*α*12/13‐RhoA pathway and inhibits the G*α*i‐cAMP/PKA pathway, thereby impeding phosphorylation of the critical eNOS activation site Ser1177 [111]. Furthermore, hyperinsulinemia downregulates miR‐21, leading to PTEN accumulation that blocks AKT‐mediated eNOS activation [112]. Additionally, xanthine oxidase (XO) activates NADPH oxidase (NOX), further suppressing eNOS activity via FoxO3a dephosphorylation [108]. The HG environment also directly promotes eNOS acetylation, impairing its catalytic function [113]. These microenvironment‐specific factors act concertedly, leading to severely compromised eNOS activity and significant reduction in NO synthesis.

**Figure 5:**
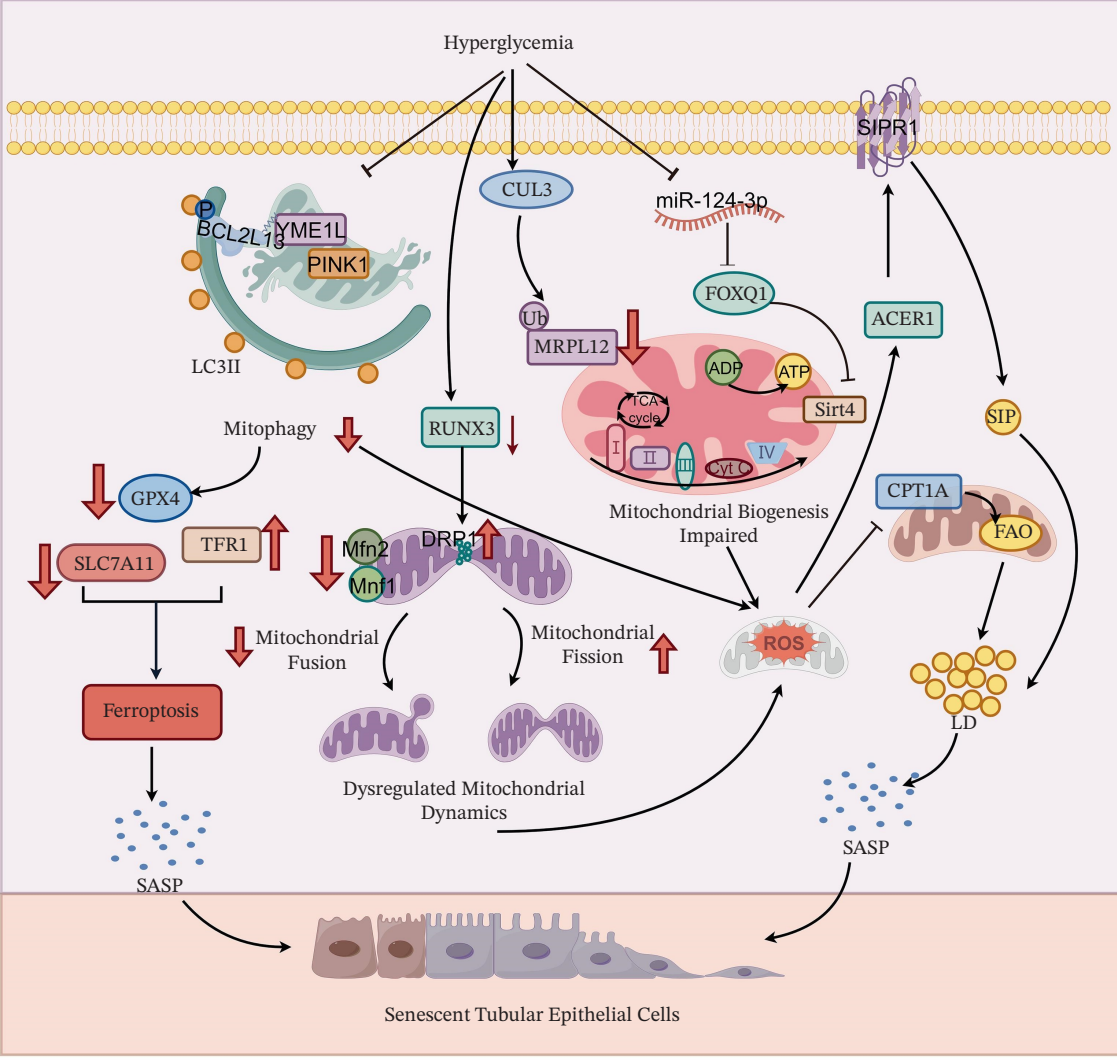
Core mechanisms of high glucose‐induced renal tubular epithelial cell senescence.

Profound suppression of the eNOS/NO axis triggers a cascade of senescent effects. First, diminished NO accompanied by elevated endothelin‐1 (ET‐1) reduces renal blood flow and induces capillary constriction [108, 109]. Second, NO deficiency downregulates expression of tight junction proteins ZO‐1 and Occludin, as well as the core glycocalyx component Syndecan‐1, directly disrupting the filtration barrier [114]. Moreover, eNOS uncoupling generates superoxide anion (O₂^−^), which synergizes with ROS derived from XO/NOX to activate the p53/p21 senescence pathway [108, 115]. Ultimately, eNOS activity inhibition reduces NO synthesis, consequently upregulating adhesion molecules VCAM‐1 and ICAM‐1 expression and promoting inflammatory infiltration [110, 114]. The resulting inflammatory microenvironment further accelerates SASP release, establishing a positive feedback loop of inflammation‐senescence [115]. Therefore, targeting eNOS activation or inhibiting its upstream regulators may effectively block this cascade [108, 111, 113, 116], highlighting the central role of this pathway in intervening against endothelial senescence (Figure 5).

### 3.4. Senescence of Renal TECs: An Engine of Disordered Metabolic Reprogramming and the Fibrotic Microenvironment

Renal tubular epithelial cells (RTECs), which exhibit relatively high metabolic and renewal activity, perform core physiological functions including solute reabsorption and metabolic waste excretion. However, this high activity level also renders them more susceptible to replicative senescence or stress‐induced premature senescence under persistent metabolic stresses like hyperglycemia and hyperlipidemia. Their high mitochondrial density and intense metabolic activity make RTECs prime targets for energy stress and oxidative damage in the DKD microenvironment characterized by HG, lipid overload, and excessive protein reabsorption [33, 117, 118]. In contrast to glomerular cells, RTEC senescence is distinctly characterized by disrupted metabolic reprogramming and a shift toward a pro‐fibrotic phenotype, directly driving renal interstitial inflammation and fibrosis processes [119, 120].

The HG microenvironment drives RTEC senescence, ultimately promoting fibrosis through three interconnected networks. **Collapse of Mitochondrial Quality Control (MQC) Network:** The HG environment triggers mitophagy deficiency characterized by downregulation of PINK1, inhibits mitochondrial biogenesis via the miR‐124‐3p/FOXQ1/Sirt4 axis and CUL3‐mediated ubiquitination and degradation of MRPL12, and induces excessive mitochondrial fission mediated by Drp1 due to RUNX3 degradation, ultimately leading to a vicious cycle of “fragmentation‐dysfunction.” **Ferroptosis Trigger:** Mitochondrial damage results in a burst of mtROS and disrupts lipid metabolism by inhibiting CPT1A activity and activating the S1P/S1PR1 signaling axis. This, combined with the functional inhibition of GPX4 caused by SLC7A11 downregulation, collectively induces ferroptosis, directly promoting cellular senescence. **SASP Bridging Role:** Senescent RTECs secrete high levels of factors such as TGF‐*β* and IL‐6, forming the SASP. This activates fibroblasts via paracrine signaling and induces EMT through autocrine mechanisms, thereby tightly coupling the intrinsic cellular senescence crisis with the interstitial fibrosis process. These networks intertwine, ultimately leading to cell cycle arrest and the malignant progression of renal interstitial fibrosis. The figure was generated with the assistance of Figdraw (http://www.figdraw.com/).

#### 3.4.1. Metabolic Overload and Mitochondrial Damage: The Initiating Hub of Aging

Under HG or HF conditions, metabolic overload in RTECs predisposes them to MQC network collapse, thus promoting cellular senescence. This collapse arises from profound dysregulation of three core mechanisms: mitophagy, biogenesis, and dynamics. Impaired mitophagy is characterized by reduced expression of key regulators YME1L and PINK1 [119, 121], leading to inadequate phosphorylation of the autophagy receptor BCL2L13 [119] and disruption of PINK1‐OPTN pathway‐mediated mitochondrial engulfment [121], resulting in damaged mitochondrial accumulation. Compromised biogenesis involves HG‐induced upregulation of CUL3, which promotes K63‐linked ubiquitination and degradation of mitochondrial ribosomal protein MRPL12 [122]. Simultaneously, miR‐124‐3p downregulation suppresses mitochondrial biogenesis via the FOXQ1‐Sirt4 axis [123]. Mitochondrial dynamics imbalance, triggered by RUNX3 degradation, causes reduced fusion proteins (Mfn1/Mfn2) and increased fission protein Drp1 [124]. Concomitant MFN1 dysfunction further attenuates mitochondrial complementation repair [125], ultimately leading to fragmented mitochondrial accumulation, membrane potential loss, and excessive mitochondrial permeability transition pore (mPTP) opening [125]. These processes are interconnected: diminished biogenesis shrinks the healthy mitochondrial pool, defective mitophagy hampers damaged organelle clearance, and dynamics imbalance accelerates fragmentation. Together, they create a vicious cycle of “fragmentation–dysfunction–accumulation,” culminating in comprehensive MQC failure.

MQC failure further aggravates senescence via aberrant metabolic reprogramming and ferroptosis. Mitochondrial dysfunction induces mitochondrial reactive oxygen species (mtROS) burst [117, 126], disrupts cristae structure, and inhibits CPT1A activity—a key enzyme in FAO. It also facilitates abnormal sphingosine‐1‐phosphate (S1P) deposition through the ACER1‐S1PR1 axis [117], promoting lipid droplet accumulation and peroxidation. Mitophagic deficiency contributes to GPX4 degradation, which synergizes with downregulated SLC7A11 and upregulated TFR1 to induce ferroptosis [127]. This process activates SASP via proinflammatory factor release such as IL‐6 and TGF‐*β*, thereby accelerating renal interstitial fibrosis [128]. Notably, targeted interventions like Astragaloside IV (AS‐IV) can restore mitochondrial function by recovering CPT1A‐mediated HSD17B10 succinylation [126], whereas selenium antioxidant nanodrug (AAN) suppresses ferroptosis by enhancing GPX4 activity [129]. This evidence underscores the significance of targeting this pathway to delay RTEC senescence.

#### 3.4.2. SASP: The Critical Bridge Linking Cellular Senescence to Interstitial Fibrosis

The core pathogenic mechanism of RTEC senescence extends beyond cell cycle arrest to encompass robust SASP factor secretion, which transforms intracellular crisis into detrimental signals that disrupt the microenvironment. Senescent RTECs release high levels of inflammatory and pro‐fibrotic mediators such as TGF‐*β* and IL‐6, which activate renal interstitial fibroblasts paracrinely, promoting their proliferation and differentiation into myofibroblasts, thereby directly driving excessive ECM deposition [12, 120].

It is particularly noteworthy that an HG environment predisposes RTECs to epithelial–mesenchymal transition (EMT) [130], while SASP provides a conducive microenvironment for this process [120]. Factors like TGF‐*β* induce phenotypic transformation in adjacent RTECs through autocrine and paracrine pathways, endowing them with migratory capacity and enabling their direct contribution to myofibroblast formation. This process is closely associated with the deubiquitinating enzyme USP11, which stabilizes TGF‐*β* receptor 2 (TGF*β*R2) and potentiates TGF‐*β* signaling [131].

Further studies elucidate that senescence propels fibrotic progression by altering cellular secretome phenotypes. For instance, BRG1‐induced senescence modifies renal tubular cell secretome, fostering fibroblast proliferation and activation [132]. Conversely, PAR2 deficiency alleviates both EMT and fibrosis by improving FAO and suppressing SASP secretion [12]. Therefore, senescent RTECs, via SASP, amplify their inherent EMT susceptibility, coupling internal metabolic collapse with external fibrotic responses [12, 133], ultimately establishing themselves as a central driving mechanism for renal interstitial fibrosis in DKD.

## 4. Clinical Translation of Targeting Cellular Senescence Mechanisms: Potential and Perspectives of Novel Renal Protective Drugs

As discussed, hyperglycemia drives heterogeneous senescence programs in renal cells by activating a complex, interwoven network of pathological mechanisms—including oxidative stress, metabolic dysregulation, chronic inflammation, and fibrosis—ultimately leading to progressive renal function decline. Notably, the multifaceted renoprotective effects of novel agents that have achieved breakthrough progress in DKD treatment in recent years, which extend beyond traditional glycemic or blood pressure control, demonstrate profound intersection with the core senescence drivers outlined above. This section explores whether and how three drug classes—sodium‐glucose cotransporter 2 inhibitors (SGLT2i), GLP‐1 receptor agonists (GLP‐1RA), and the nonsteroidal MR antagonist finerenone—may retard DKD progression by intervening in key cellular senescence pathways.

### 4.1. SGLT2 Inhibitors: Alleviating Metabolic Overload and Energy Crisis With the Potential to Modulate Senescent Phenotypes

The CREDENCE trial confirmed the renal protective effects of canagliflozin in patients with chronic kidney disease, including those with diabetes [134]. Furthermore, the DAPA‐CKD study demonstrated that dapagliflozin provides significant benefits in patients with chronic kidney disease and diabetes [135]. The EMPA‐KIDNEY trial confirmed the long‐term renal benefits of empagliflozin in patients with chronic kidney disease (including those with DKD, accounting for up to 31% of participants), with protective effects persisting for up to 12 months even after drug discontinuation [136].

A 2024 systematic review explicitly proposed that SGLT2i possess potential to target kidney aging pathways [137]. Recent primary research has provided robust molecular‐level evidence supporting this view, revealing their multi‐target anti‐aging mechanisms. First, dapagliflozin was found to promote the generation of ketone bodies (*β*‐hydroxybutyrate). The latter activates the antioxidant transcription factor NRF2, significantly reducing the expression of key senescence markers (p16^INK4a^, p21, and p53) in DKD models, alleviating oxidative stress and DNA damage. Notably, this effect is independent of its glucose‐lowering action [138]. Second, SGLT2 inhibitors can directly upregulate the expression of the “longevity protein” Klotho. Clinical studies have confirmed that serum and urinary Klotho levels are significantly elevated in patients with DKD treated with drugs like canagliflozin, and this elevation is associated with reduced inflammation, providing direct evidence for the anti‐aging effects of SGLT2i in humans [139]. From a mechanistic perspective, early research has confirmed that the HG environment induces senescence in renal tubular cells via an SGLT2‐dependent pathway [140]. Dapagliflozin, by reducing glucose influx, can block the subsequent ROS burst‐ATM/p53‐p21 signaling cascade, thereby preventing senescence in renal TECs [141]. Additionally, studies have found that canagliflozin can suppress the HG‐upregulated Hedgehog‐interacting protein (HHIP)‐mediated senescent phenotype in renal tubular cells [142]. These findings from cell and animal models provide a basis for initiating prospective clinical studies specifically designed to evaluate the impact of SGLT2i on kidney senescence biomarkers. SGLT2i may emerge as a novel therapeutic strategy for DKD by intervening in senescence programs.

### 4.2. GLP‐1 Receptor Agonists: From Metabolic Regulation to Potential Pleiotropic Renal Protection

The renoprotective effects of GLP‐1RAs may partially stem from their antagonism of key pathological environments that drive cellular senescence. Although direct evidence for their regulation of classical senescence markers like p16/p21 is still accumulating, GLP‐1RAs have been confirmed to remodel the senescent microenvironment at multiple levels. First, their potent anti‐inflammatory effects directly target the SASP. Liraglutide can inhibit chronic renal inflammation and aberrant macrophage activation by downregulating the RAGE [143]. Furthermore, both liraglutide and semaglutide can suppress the NLRP3 inflammasome, reducing the release of key SASP factors such as IL‐1*β* [144]. Second, GLP‐1RAs can ameliorate the metabolic and oxidative stress that trigger senescence. Dulaglutide can upregulate the antioxidant protein GPX4 and inhibit the STAT3 pathway, thereby alleviating renal oxidative damage and inflammation [145]. Exenatide, on the other hand, enhances mitochondrial function by activating the AMPK pathway and inhibits lipotoxicity‐associated cellular damage [146]. Therefore, GLP‐1RAs likely indirectly delay HG‐induced renal cellular senescence primarily by curbing chronic inflammation (the core of SASP) and mitigating metabolic/oxidative stress, two critical upstream drivers.

It should be noted that current primary research evidence on the direct regulation of core senescence programs by GLP‐1RAs in DKD remains relatively limited. Existing evidence focuses more on elucidating their effects against upstream pathological drivers of senescence, such as inflammation and oxidative stress. Consequently, future research employing cellular senescence reporter systems or in‐depth assessment of senescence biomarkers to directly validate the specific impact of GLP‐1RAs on the senescent phenotype of different renal parenchymal cells under high‐glucose conditions will be crucial. This direction is key to fully elucidating their complete renoprotective mechanisms and confirming their potential as a “senotherapeutic” strategy.

### 4.3. Finerenone: Targeting Inflammatory Senescence and Disrupting the Senescence–Fibrosis Nexus

Finerenone, a novel nonsteroidal MR antagonist, has been established as a cornerstone in the modern comprehensive management of DKD [147]. Its remarkable renal protective efficacy has been confirmed in large‐scale clinical trials such as FIDELIO‐DKD [148]. Although molecular mechanistic research directly investigating the impact of finerenone on classical senescence markers (e.g., p16, p21) in renal cells is still in its early stages, based on its well‐defined pharmacological target, the benefits of finerenone in retarding DKD progression are believed to be closely related to its intervention in the core pathological process of “inflammatory aging.”

Mechanistically, the core action of finerenone lies in its potent antagonism of the MR. Within the DKD environment characterized by hyperglycemia and activation of the renin–angiotensin–aldosterone system, excessive MR activation serves as a key hub driving renal oxidative stress, chronic inflammation, and fibrosis [147]. This chronic inflammatory microenvironment is precisely the primary driver that induces and sustains cellular senescence, particularly the SASP [147, 149]. Therefore, by source—specifically blocking the MR signaling pathway, finerenone powerfully suppresses local renal inflammation and the fibrotic process. This action directly targets the very milieu from which SASP arises, potentially indirectly curbing the harmful secretory effects of senescent cells and their paracrine propagation. Consequently, it may mitigate the persistent damage to key parenchymal cells such as glomerular podocytes and renal TECs, thereby slowing the decline in tissue function [150]. Additionally, its effects on improving endothelial function and alleviating oxidative stress also help eliminate the initial pressures that trigger cellular senescence.

It is important to note that, similar to the case with GLP‐1RAs, primary research evidence directly elucidating how finerenone regulates core senescence programs in DKD models remains very limited. Current understanding is primarily based on the correlation between its clinical anti‐inflammatory and anti‐fibrotic efficacy and the theoretical framework of cellular senescence. Future research urgently needs to directly validate the effects of finerenone intervention on senescence markers (e.g., SA‐*β*‐gal activity, p16^INK4a^ expression) and SASP factor profiles in renal cells under high‐glucose conditions, using animal models and cell experiments. This will provide empirical evidence for its “senescence‐modulating” potential and deeply reveal the intersection points between its traditional anti‐fibrotic actions and the senescence axis.

## 5. Conclusion

In summary, during DKD progression, although the high‐glucose microenvironment serves as a common initiating factor triggering senescence across various renal cell types, and the terminal senescent state universally presents as irreversible cell cycle arrest and SASP secretion, the underlying driving mechanisms, core pathways, and pathological contributions exhibit distinct cell‐type specificity (as summarized in Table 1).

**Table 1:** Senescence mechanisms of key renal cells in diabetic kidney disease: Heterogeneous features and pathological outputs.

Cell type	Core senescence mechanisms	Key signaling nodes and molecular features	Major pathological outputs and functional impacts
Podocyte	Unique metabolic crisis Lipotoxic injury Energy homeostasis exhaustion Dysfunction of precise structure‐function hubs Impaired mechanical stress sensing	Dysregulated GSK3*β* signaling Dysfunction of the GPR124‐Vinculin‐FAK axis mTORC1/AMPK signaling imbalance cGAS‐STING pathway activation	Foot process effacement, Filtration barrier disruption, Proteinuria
Mesangial cell	Pathological metabolic reprogramming Glycolytic shift NOX4‐mediated oxidative stress Vicious cycle of “senescence and fibrosis”	Aberrant activation of the STAT5 signaling axis Caveolin‐1 dysfunction Impaired autophagy Elevated expression of TGF‐*β*, etc.	Mesangial expansion, Glomerulosclerosis
Glomerular endothelial cell	Glycocalyx barrier disruption Dysfunctional eNOS/NO signaling axis Microcirculatory disturbance	ENOS uncoupling ASK‐1‐p38 pathway activation CXCR2/NF‐*κ*B pathway activation Upregulation of VCAM‐1/ICAM‐1	Loss of fenestrae, Capillary constriction, Microcirculatory dysfunction, Inflammatory infiltration
Tubular Epithelial Cell	Collapse of mitochondrial quality control Ferroptosis Secretion of pro‐fibrotic SASP	Deficiency of key factors (e.g., PINK1/Parkin) mtROS burst GPX4 degradation GF‐*β*/Smad pathway activation	Tubular injury, EMT, Inflammation, Interstitial fibrosis

The hallmark of podocyte senescence lies in its unique metabolic crisis, primarily characterized by lipotoxic injury and exhaustion of energy homeostasis, coupled with dysfunction of sophisticated structural‐functional hubs. This involves aberrant signaling of key molecules such as GSK3*β* and GPR124, ultimately leading to filtration barrier collapse and proteinuria development.

MC senescence, in contrast, is prominently featured by pathological metabolic reprogramming, with a notable shift towards glycolysis. This is accompanied by NOX4‐mediated oxidative stress and a “senescence–fibrosis” malignant cycle driven by coordinated action of the STAT5 and Caveolin‐1 signaling axes, thereby positioning these cells as critical promoters of glomerulosclerosis.

The uniqueness of GEC senescence stems from structural disintegration of the glycocalyx barrier and functional disruption of the eNOS/NO signaling axis, which subsequently induces microcirculatory dysfunction and a vascular senescence phenotype.

RTEC senescence, however, is triggered by comprehensive MQC network collapse. This collapse initiates cascading events including ferroptosis, SASP release, and EMT, collectively acting as the core engine driving renal tubulointerstitial fibrosis.

This mechanistic heterogeneity originates from inherent differences in anatomical location, physiological function, and molecular regulatory networks among distinct cell types. It is noteworthy that the renoprotective effects of novel drugs such as SGLT2 inhibitors, GLP‐1RAs, and finerenone may partly involve intervention in the aforementioned senescence drivers. For instance, evidence is relatively clear that SGLT2 inhibitors delay cellular senescence by alleviating metabolic overload and oxidative stress. Furthermore, the anti‐inflammatory action of GLP‐1RAs and the anti‐fibrotic effect of finerenone demonstrate profound intersection with the senescence‐related inflammatory and fibrotic microenvironment. However, whether these two drug classes directly and specifically delay the senescence programs of different renal cell types requires future research to provide direct evidence.

A deeper understanding of both the commonalities and heterogeneity within this cellular senescence signaling network is of paramount importance for developing cell‐type‐specific, precise anti‐senescence therapeutic strategies.

## 6. Discussion and Perspectives

This article systematically elucidates the specific molecular mechanisms and pathway heterogeneity underlying the senescence of four key renal cell types—podocytes, MCs, GECs, and RTECs—driven by the HG microenvironment in DKD. The origin of these specific senescence programs lies in the inherent biological properties of each cell type, particularly the vast disparity in their proliferative and renewal capacities: from terminally differentiated podocytes with almost no self‐renewal ability to RTECs possessing relatively strong replicative potential. This intrinsic difference fundamentally shapes their thresholds of tolerance to hyperglycemic damage, repair capabilities, and pathways toward senescence [10, 151, 152]. Although the initiation mechanisms and core pathways of senescence differ among these cell types, they do not operate in isolation. Instead, through extensive cross‐talk and paracrine actions, they form intricate senescent networks within the organ, generating amplifying effects. For instance, podocyte and endothelial cell senescence influence each other via dysregulation of the VEGF signaling pathway [153, 154]. Communication between MCs and RTECs primarily relies on exosomes/extracellular vesicles (EVs), synergistically promoting inflammatory responses, fibrosis, and apoptosis in DKD, thereby driving the initiation and progression of both glomerular and tubulointerstitial pathologies [155]. Moreover, signals such as lipotoxicity, oxidative stress, and fibrosis form positive feedback loops across different cell types. Therefore, future research needs to further integrate a multicellular interaction perspective to decipher the dynamic construction and evolution of senescent micro‐networks at the organ and even systemic levels.

From a translational medicine perspective, targeting cellular senescence has emerged as a promising strategy for DKD intervention: senolytics can selectively clear senescent cells [20], while senomorphics can suppress the detrimental effects of the SASP [9]. However, given the high heterogeneity of senescence mechanisms, future therapeutic strategies must be further refined and directed towards specific cell types. For example, interventions targeting GSK3*β* or GPR124 in podocytes, STAT5 or Caveolin‐1 in MCs, the eNOS/NO axis in GECs, and the MQC‐ferroptosis pathway in RTECs hold promise for achieving more specific and less toxic therapeutic outcomes. Concurrently, developing biomarkers and imaging techniques capable of distinguishing between different senescent cell types is a crucial prerequisite for personalized therapy. Notably, the pleiotropic mechanisms of action of novel drugs that have demonstrated outstanding renal and cardiovascular protective effects in DKD clinical practice (e.g., SGLT2 inhibitors, GLP‐1 receptor agonists, and finerenone) are being revealed to intersect profoundly with senescence regulatory networks. These drugs may indirectly delay or modify the senescence process in different renal cells by intervening upstream in core drivers of senescence, such as metabolic overload, chronic inflammation, and oxidative stress. This suggests that future anti‐senescence strategies are not limited to directly targeting senescent cells themselves but may also achieve systemic protection through the earlier intervention concept of “reconstructing the senescent microenvironment,” utilizing existing or novel multi‐target drugs.

Although targeting cellular senescence holds broad prospects for DKD treatment, the translation from basic research to clinical application still faces a series of key challenges. First, the majority of mechanistic evidence originates from animal or cell models, and its generalizability to human DKD kidney tissue requires further validation. Future efforts should focus on systematically mapping the expression profiles of p16^INK4a^, p21^CIP1^, SA‐*β*‐gal activity, and cell type‐specific SASP factors in human renal biopsy samples, precisely distinguishing them from mechanisms such as apoptosis, to establish a high‐resolution atlas of senescent cells in human DKD. Second, the dynamic changes of renal cellular senescence across different stages of DKD (e.g., early, progressive, advanced) remain unclear, and the long‐term safety, delivery efficiency, and tissue specificity of interventional strategies like senolytics still need improvement.

Particularly crucial is the lack of conclusive evidence regarding whether drugs with proven renal benefits—such as SGLT2 inhibitors, GLP‐1 receptor agonists, and finerenone—directly and specifically intervene in the senescence programs of different renal cell types. Future research needs to utilize technologies like senescence reporter systems, single‐cell transcriptomics, and spatial proteomics to directly validate the impact of these drugs on specific senescence markers and cell type‐specific SASP profiles in preclinical models and patient samples. Examples include clarifying whether SGLT2 inhibitors preferentially protect podocytes via the ketone body‐NRF2 axis, whether GLP‐1RAs can reverse tubular metabolic senescence through the AMPK pathway, and whether finerenone effectively disrupts the “senescence–fibrosis” association in MCs by inhibiting MR signaling.

Finally, a core direction for future translational research is how to effectively integrate senescence‐related molecular indicators with clinical kidney disease staging and prognostic assessment tools, constructing a biomarker system to guide individualized treatment and achieving genuine “precision nephrology.”

In conclusion, in‐depth research into the heterogeneous mechanisms of renal cellular senescence not only contributes to a deeper understanding of the multidimensional pathogenesis of DKD but also provides important directions for developing novel targeted therapies. Through interdisciplinary collaboration and leveraging cutting‐edge technologies such as single‐cell omics, gene editing, and advanced drug delivery systems, the future holds promise for achieving precise molecular subtyping and therapeutic breakthroughs in DKD based on cellular senescence signatures.

**Nomenclature**
AGEs: advanced glycation end products
*α*‐SMA: *α*‐smooth muscle actin
Ang II: angiotensin II
AS‐IV: astragaloside IV
AAN: antioxidant nanodrug
Cav‐1: caveolin‐1
DDR: DNA damage response
DKD: diabetic kidney disease
DAMPs: damage‐associated molecular patterns
ESRD: end‐stage renal disease
ERS: endoplasmic reticulum stress
ECM: extracellular matrix
eGlx: endothelial glycocalyx layer
eNOS: endothelial nitric oxide synthase
ET‐1: endothelin‐1
EMT: epithelial–mesenchymal transition
EVs: exosomes/extracellular vesicles
FPs: foot processes
FFAR4: free fatty acid receptor 4
FAK: focal adhesion kinase
FAO: fatty acid oxidation
GBM: glomerular basement membrane
GFR: glomerular filtration rate
GECs: glomerular endothelial cells
GSK3*β*
: glycogen synthase kinase 3*β*
GLP‐1RA: glucagon‐like peptide‐1 receptor agonists
HG: high glucose
HPSE: heparanase
HYAL1: hyaluronidase 1
HHIP: hedgehog‐interacting protein
mtDNA: mitochondrial DNA
MCs: mesangial cells
MQC: mitochondrial quality control
MMP2: matrix metalloproteinase 2
mPTP: mitochondrial permeability transition pore
mtROS: mitochondrial reactive oxygen species
MR: mineralocorticoid receptor
MQC: mitochondrial quality control
NO: nitric oxide
ROS: reactive oxygen species
Rb: retinoblastoma
RTECs: renal tubular epithelial cells
PKM2: pyruvate kinase M2
SD: slit diaphragm
SDC4: syndecan‐4
SASP: senescence‐associated secretory phenotype
S1P: sphingosine‐1‐phosphat
SGLT2i: sodium‐glucose cotransporter 2 inhibitors
TECs: tubular epithelial cells
TGF*β*R2: TGF‐*β* receptor 2
NOX: NADPH oxidase
NOX4: NADPH oxidase 4
UPR: unfolded protein response
XO: xanthine oxidase

## Author Contributions

**Jing Wang and Tian Liu:** writing – original draft, conceptualization, investigation. **Ni Lin:** investigation. **Qi Wang:** investigation, visualization. **Kai Zhu:** investigation, visualization. **Beijia Xu:** investigation, visualization. **Xiaomin Wang:** writing – review and editing, supervision, funding acquisition. Jing Wang and Tian Liu contributed equally to this work.

## Funding

This study was supported by Sichuan Provincial Science and Technology Program, 2024YFFKO148; the 2025 National Natural Science Foundation Cultivation Project of Affiliated Hospital of Chengdu University of Traditional Chinese Medicine, 2025NSFCPY042, 2025NSFCPY040; the Potential Talents of “Hundred Talents Program” for Enhancement of Scientific Research Ability in the Affiliated Hospital of Chengdu University of Traditional Chinese Medicine, 22‐Q36.

## Conflicts of Interest

The authors declare no conflicts of interest.

## Data Availability

The data that support the findings of this study are available within the article.
